# Concurrent endoscopic ultrasound-guided biliary drainage and ultra-slim (6.5-French) cholangioscopy for malignant biliary obstruction: A one-procedure technical innovation

**DOI:** 10.1055/a-2879-0393

**Published:** 2026-06-23

**Authors:** Shan-Shan Hu, Wei-Hui Liu

**Affiliations:** 1 Department of Gastroenterology and Hepatology 89669 Sichuan Provincial People's Hospital, School of Medicine, University of Electronic Science and Technology of ChinaChengduSichuanChina

Endoscopic ultrasound (EUS)-guided biliary drainage (EUS-BD) and peroral cholangioscopy are conventionally staged procedures due to fistula maturation requirements. 1​ 2​ ​3​ We report a novel “single-session” approach using a 6.5Fr ultra-slim cholangioscope immediately during EUS-BD. This protocol not only enhances the success rate of EUS-BD and reduces bile leakage but also saves time by eliminating the waiting interval.

**Fig. 1:**
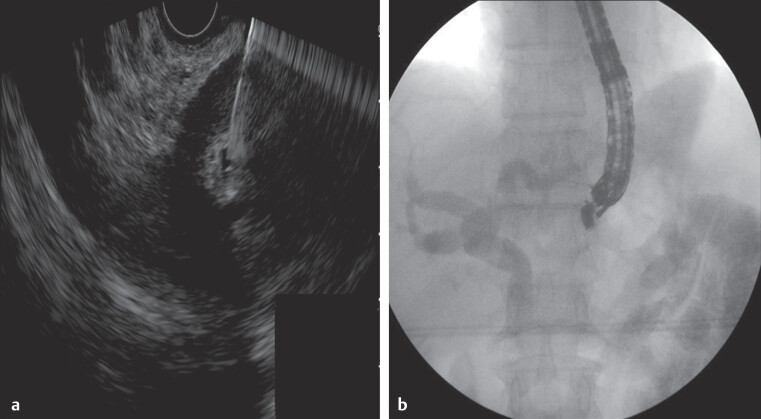
EUS-BD was performed via a transgastric approach to B3.

**Fig. 2:**
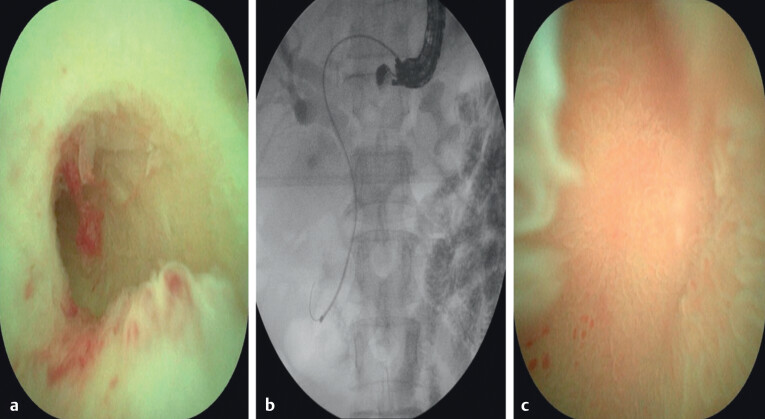
The “single-session” technical innovation achieves integrated diagnosis and therapy. ( **a** ) Direct visualization confirmed the malignant stricture at the distal bile duct. ( **b** ) Under cholangioscopic guidance, the guidewire was successfully advanced across the stricture segment. ( **c** ) Direct visualization confirmed no distal obstruction in the duodenal cavity.

**Fig. 3:**
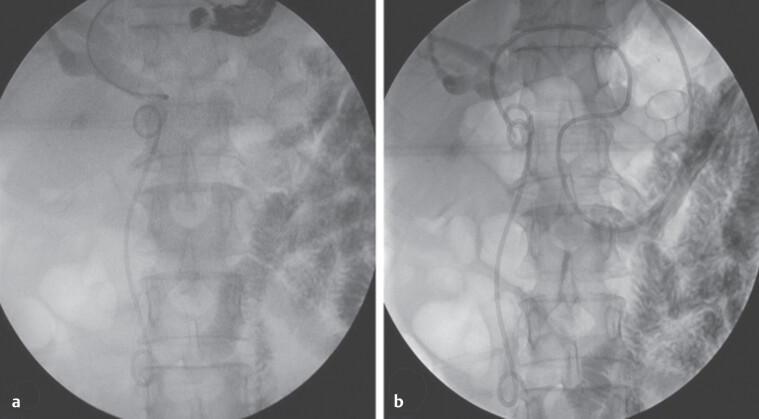
Combined biliary drainage: ( **a** ) EUS-AS and ( **b** ) EUS-HGS.

A 58-year-old man with unresectable pancreatic head cancer presented with obstructive jaundice. Endoscopic retrograde cholangiopancreatography failed due to duodenal infiltration by tumors. EUS-BD was performed via a transgastric approach to B3 ( Fig. 1 ), creating a hepaticogastrostomy with 6 mm balloon dilation. Immediately thereafter, a 6.5Fr digital single-operator cholangioscope (eyeMAX; Micro-Tech, Nanjing, China) with a 0.75-mm working channel and 220-cm length was advanced through the fresh fistula without fluoroscopic guidance. Direct visualization confirmed a malignant stricture at the distal bile duct. Under cholangioscopic guidance, the guidewire was successfully advanced across the stricture segment and antegrade into the duodenal lumen ( Fig. 2 ). Following the direct visual confirmation of no distal obstruction in the duodenal cavity, we subsequently performed EUS-guided antegrade stenting combined with EUS-guided hepaticogastrostomy to achieve combined biliary drainage ( Fig. 3 ). The total procedure time was 35 minutes. No bile leakage, bleeding, or perforation occurred. Bilirubin normalized within 9 days ( Video 1 ).

**Video 1** Concurrent endoscopic ultrasound-guided biliary drainage and ultra-slim (6.5-French) cholangioscopy for malignant biliary obstruction: A one-procedure technical innovation.


Single-session EUS-BD with 6.5Fr cholangioscopy is technically feasible and safe for malignant biliary obstruction. The ultra-slim profile enables immediate fistula traversal, accelerating diagnosis and therapy.

Endoscopy_UCTN_Code_TTT_1AS_2AH
